# Ecological Factors at Fine Spatial Scale Associated With Habitat Use by Tigers in Western Terai Arc Landscape, Nepal

**DOI:** 10.1002/ece3.71109

**Published:** 2025-03-13

**Authors:** Shyam Kumar Shah, Jhamak Bahadur Karki, Balram Bhatta, Naresh Subedi, Rabin Bahadur K. C., Rabin Kadariya, Ajay Karki, Umesh Paudel, Babu Ram Lamichhane, Arjun Thapa

**Affiliations:** ^1^ Agriculture and Forestry University (AFU) Rampur Nepal; ^2^ Department of National Parks and Wildlife Conservation (DNPWC) Kathmandu Nepal; ^3^ National Trust for Nature Conservation (NTNC) Lalitpur Nepal; ^4^ University of Wyoming Laramie Wyoming USA; ^5^ USAID Biodiversity (Jal Jangal) Lalitpur Nepal; ^6^ Institute of Zoology Chinese Academy of Science Beijing China; ^7^ Institute of Fundamental Research and Studies (InFeRS) Kathmandu Nepal

**Keywords:** Bardia–Banke complex, camera trapping, habitat use, prey index, waterholes

## Abstract

Conservation of designated source sites is a fundamental strategy for global tiger recovery. Reliable estimates of tiger *Panthera tigris* habitat use within these source sites are crucial for informing effective management strategies. In this study, we assessed tiger habitat use within the Bardia‐Banke Complex, one of the 42 global source sites, situated in the western Terai Arc Landscape (TAL) of Nepal. We conducted a grid‐based detection and non‐detection camera trap survey across 719 grid cells, each measuring 2 × 2 km^2^. To assess tiger habitat use while accounting for imperfect detectability, we applied a single‐season occupancy model. We analyzed nine covariates that have the potential to influence tiger habitat use in the Complex, including terrain, co‐predators, prey, water availability, and disturbance. We found that fine scale (2 × 2 km^2^) tiger habitat use in the Complex was 0.43 (SE ± 0.0085, 95% CI: 0.414–0.448). Our analysis demonstrated that tigers used habitats unevenly across the Bardia‐Banke Complex. Our results showed that the terrain ruggedness index, prey index, and proximity to waterholes were key determinants of tiger habitat use. Tiger habitat use was positively associated with prey abundance and negatively associated with terrain ruggedness and distance to waterholes. We emphasize the importance of influencing habitat covariates that determine the probability of habitat use for taking appropriate habitat‐management decisions for tiger conservation in the TAL. We highlight the importance of periodic assessment of tiger habitat use in this globally significant source site to monitor changes in spatial habitat use patterns, serving as a measure of the effectiveness of wildlife management interventions.

## Graphical abstract

Systematic camera trapping at fine scale revealed that tiger habitat use was positively associated with prey abundance and negatively associated with terrain ruggedness and distance to waterholes in Bardia –Banke Complex in Nepal.

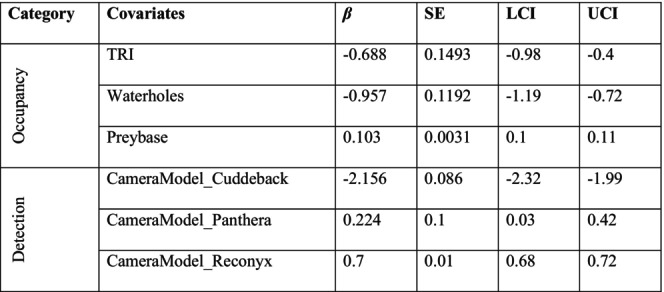

## 1 Introduction

**FIGURE 1:**
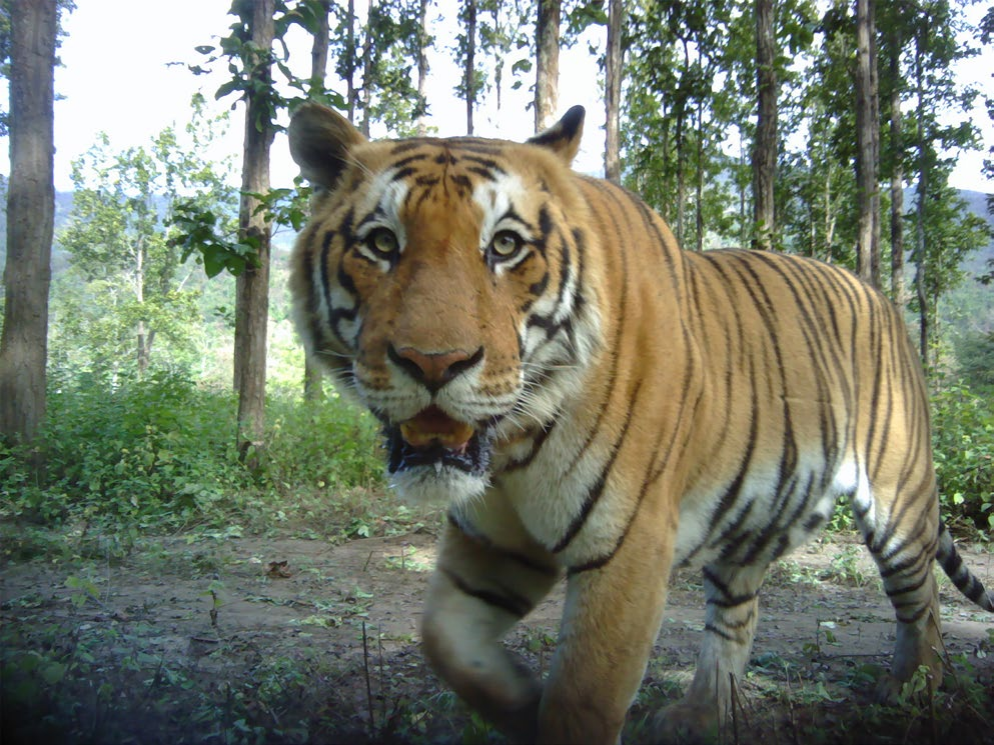
Camera trap detection photograph of a tiger in the study area.

Large carnivores have experienced substantial population decline and range contraction across the globe (di Minin et al. 2016; Wolf and Ripple 2017). They play a crucial ecological role, exerting cascading effects through predation that influence entire ecosystems and regulate trophic dynamics (Terborgh et al. 2001; Wolf and Ripple 2016; Gray et al. 2023). The tiger *Panthera tigris* (Figure 1), a wide‐ranging big cat native to the forests and grasslands of south and southeast Asia, the Russian Far East, and northeast China (Forrest et al. 2011), is now confined to 10 countries, including Nepal (GTRP 2023). Tigers have experienced a significant decline in both population and range. Tigers, Asia's most iconic carnivore, are currently restricted to less than 10% of their historic range and have experienced recent extinction from three countries in mainland Southeast Asia (Gray et al. 2023; Sanderson et al. 2023) with an estimated population of 3726–5578 individuals (Goodrich et al. 2022). Most of the global tiger population is concentrated within approximately 42 “source sites.” The protection and effective management of tiger populations in these source sites constitute the cornerstone of global tiger conservation strategies (Walston et al. 2010).

Human‐induced activities have caused the loss and degradation of wildlife habitats, accelerating the probability of the extinction of many wildlife species globally (Carter et al. 2013). Developmental activities could cause modification of landscape forms, induce various disturbances in terms of dispersal and gene flow, which are serious challenges to wide‐ranging species (Milda et al. 2023). Land use change affects the distribution, abundance, and behavioral patterns of the mega species, either causing local extinctions or inducing human‐wildlife conflict (Milda et al. 2023). Many species have already disappeared from their historical ranges due to habitat loss and fragmentation by agricultural expansion, linear infrastructure development, overexploitation of natural resources, decreased prey abundance, diseases, poaching, and illegal trade (Ripple et al. 2015; Wolf and Ripple 2017). Therefore, understanding the ecological requirements of a species is crucial for long‐term conservation of the species in the particular habitat state.

Protected Areas (PAs) have been crucial for tiger conservation, providing secure habitats for housing source populations (Forrest et al. 2011). Due to large home ranges, tiger conservation strategies require a landscape‐scale approach (Wikramanayake et al. 2011); sole protected area conservation is inadequate. Terai Arc landscape (TAL), an important conservation landscape for megafauna, especially for tigers along the southern border of Nepal with India (Wikramanayake et al. 2011), has faced severe anthropogenic pressure and land use changes (Lamichhane et al. 2021; DNPWC and DFSC 2022; Thapa et al. 2022). Bardia–Banke Complex (hereafter; BB Complex), which includes Bardia National Park, one of the tiger source sites, and Banke National Park, with adjoining forests, is recognized as one of the prime habitats for tigers in the TAL (DNPWC and DFSC 2022). BB Complex holds 42.2% (150 of 355 individuals) of the total tiger population of Nepal. As the tiger population in the BB Complex gradually increases, it becomes imperative to evaluate the ecological and anthropogenic factors influencing their habitat use. A deeper understanding of these factors is crucial, given the current knowledge gaps in this area.

Large carnivores' occupancy is highly influenced by the habitat type, vegetation type, degree of human disturbance (Karanth et al. 2006), abundance of suitable wild prey species (Wolf and Ripple 2016), and degree of habitat fragmentation (Tilman et al. 2017). Other factors such as forest type, vegetation cover and reserve size (Sunarto et al. 2015; Havmøller et al. 2019), livestock depredation, and human settlements (Karanth et al. 2011; Harihar and Pandav 2012) are regarded as the important covariates influencing the large carnivore habitat use. However, interpreting habitat association of tigers is challenging in the small protected areas because these factors can vary across different spatial and temporal scales. Moreover, conflict between humans and wildlife often arise at the local scale, emphasizing the importance of understanding how large carnivores' habitat use varies in protected habitats at a finer scale.

In this study, we aim to assess habitat use of tigers at a fine spatial scale in the BB Complex using detection and non‐detection camera trap survey data. We assess the likely factors that influence habitat use in a single‐season occupancy framework.

## 2 Material and Methods

### 2.1 Study Area

**FIGURE 2:**
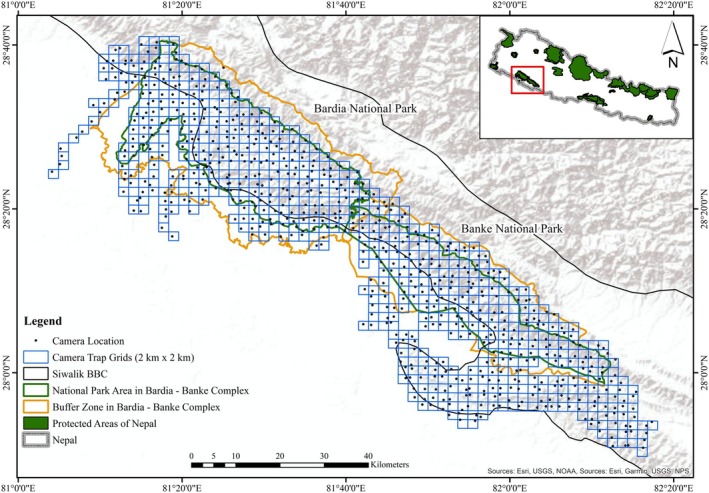
Bardia–Banke Complex includes Bardia National Park in the western section and Banke National Park in the eastern section. The map inset shows the location of the BB Complex and other protected areas within Nepal.

The Bardia‐Banke Complex comprises Bardia National Park (Bardia NP), one of the identified 42 global source sites for tigers (Walston et al. 2010), contiguous to Banke National Park (Banke NP) and surrounding forest areas (Figure 2). The Complex covers 1580 km^2^ of national parks, an 850 km^2^ buffer zone, and adjoining forests with an elevation ranging from 100 to 1500 m above sea level. The Complex is embedded within the transboundary Terai Arc Landscape (TAL), identified as one of the global priority tiger conservation landscapes (TCL) (Sanderson et al. 2023). This landscape, which spans between Nepal and India and serves as a critical habitat for sustaining the tiger's source population, has natural dispersal likely through the Khata Biological Corridor to Katarniaghat Wildlife Sanctuary and via the Kamdi Biological Corridor to Suhelwa Wildlife Sanctuary in India (Bhatt et al. 2023).

Babai River, Karnali River, and West Rapti River serve as the lifelines for the parks' biodiversity richness. Additionally, more than 50 ephemeral streams originating from the Churia hills in this Complex drain rainwater during the monsoon season and remain dry for the rest of the year. The Complex comprises a mosaic of habitats, including riverine forest, mixed hardwood forest, sal forest, Churia hill mixed forest, alluvial grassland, human‐managed grasslands, rivers, natural wetlands, and artificial waterholes. Water availability is a critical limiting factor for wildlife, especially during the dry season (Shah et al. 2024). This Complex is principally covered by tropical deciduous forests, alluvial floodplain grasslands, rivers, and both natural water sources and waterholes (BNP 2022; BaNP 2022).

BB Complex holds the largest tiger population in Nepal (150) with the highest tiger density, that is, 7.96 (SE 0.29) (Shah et al. 2024). Besides tigers, the Complex (Figure 2) harbors more than 60 species of mammals, including charismatic species like the Asian elephant ( *Elephas maximus* ), Ganges river dolphin (*Planista gangetica*), Gharial ( *Gavialis gangeticus* ), and Greater one‐horned rhinoceros ( *Rhinoceros unicornis* ). The Complex has a diverse prey species, including Spotted deer ( *Axis axis* ), Barking deer ( *Muntiacus muntjak* ), Four‐horned antelope ( *Tetracerus quadricornis* ), Sambar ( *Cervus unicolor* ), and Wild boar ( *Sus scrofa* ) (BaNP 2022; BNP 2022).

The Complex is surrounded by densely populated human settlements with an average density of ~400 people/km^2^ (CBS Nepal 2022) and agricultural fields. Forests in the Complex are intersected by various linear infrastructures, including the East–West National Highway (72 km in Bardia NP and 30 km in Banke NP), the North–South Highway between Bardia NP and Banke NP, power transmission lines, and an irrigation canal (BNP 2022; BaNP 2022).

### 2.2 Method

#### 2.2.1 Field Survey and Data Collection

We used a grid‐based sampling approach and divided the study area into 719 grid cells of size 2 × 2 km^2^ for systematic detection and non‐detection camera trap surveys. A pair of camera traps was systematically placed in all 719 grid cells across the Complex from 16 December 2021 to 12 March 2022. Cuddeback (C1), Panthera (V5 and V6) and Reconyx automated cameras with white flash were used to capture photos of tigers and other wild animals. Due to large geographic coverage and logistical constraints, the study area was divided into four blocks and surveyed subsequently. We set up camera traps in each grid at locations presumably with high tiger capture probability based on intensive sign surveys prior to camera placement. Camera traps were mounted on standing trees or on pegged wooden poles 45–60 cm above the ground, perpendicular to, and 6–10 m apart on either side of game trails, forest roads, and riverbeds without using any bait. Each camera trap had a unique camera number, and all locations were marked with the Global Positioning System (GPS). Each survey occasion consisted of a 15–16 day camera‐trapping session, with cameras monitored twice a week in accordance with Nepal's tiger and prey monitoring protocol (DNPWC 2017). Tiger detection (photographs) and non‐detection (absence of photo captures) were systematically recorded and compiled for analysis.

#### 2.2.2 Analysis of Habit Use in Single Season Occupancy Framework

We applied a single‐season occupancy model to assess habitat use of the tiger (Mackenzie 2006). It used detection and non‐detection data with two inferences: the probability of detection (*p*; the probability of photo capturing a species that is present in a grid on a single occasion) and the probability of occupancy (*ψ*; the probability that the species occupies a random site in a given period) using a maximum likelihood approach. We created a detection history of tigers in each grid, considering 24 h as a sampling occasion (Kafley et al. 2016; Ash et al. 2020). Detection of tigers in each sampling occasion was coded as ‘1’ and non‐detection as ‘0’ (Kafley et al. 2016).

We employed a two‐step model‐fitting approach to analyze detection probability and occupancy (habitat use), following MacKenzie et al. (2002) and Sunarto et al. (2012). In the first step, detection probability was modeled under the assumption of constant occupancy, as outlined by Thapa et al. (2019). The best‐performing detection model served as the basis for subsequent models that incorporated covariates to explain tiger habitat use. In the second step, occupancy was estimated using the most supported detection probability model (Karanth et al. 2011; Srivathsa et al. 2018).

Assuming random movement of animals among fine‐scale sampling sites (Kendall 1999), we relaxed the closure assumption, defining “occupied locations” as “used locations” (MacKenzie et al. 2004; Kafley et al. 2016). To evaluate the effects of covariates on habitat use, we employed single‐season occupancy models (Mackenzie 2006) using the R package ‘*unmarked*’ (Fiske and Chandler 2011; R Core Team 2022). All candidate model comparisons were based on Akaike information criterion (AIC) values (Burnham and Anderson 2002). The strength of the relationship between each covariate and the occupancy probability was assessed using the estimated *β* coefficients.

#### 2.2.3 Covariates Selection

We selected nine covariates (Table 1) that have the potential to influence tiger habitat use, including terrain ruggedness index (TRI) (Karanth et al. 2009), prey index (Barber‐Meyer et al. 2013; Harihar and Pandav 2012), carnivore index, habitat types, distance to rivers, distance to waterholes, and distance to settlements (Karanth et al. 2011; Harihar and Pandav 2012; Sunarto et al. 2012; Kafley et al. 2016).

**TABLE 1:** Covariate selection with *priori* hypotheses. The symbols denote possible influences of the covariates: “+” positive influence, “−” negative influence, and “±” positive/negative influence.

Habitat use Covariates	Types	Rationale	A priori relationship
			Psi(*ѱ*)
Occupancy covariates			
Terrain Ruggedness Index (TRI)	Continuous	Tigers were recorded in the Churia Hills (Thapa and Kelly 2017). Low TRI favor tiger and prey base	−
Distance to river	Continuous	In dry months, water becomes a critical limiting factor in the BB Complex (Thapa et al. 2023). Proximity to river favor tiger and prey base. (Kafley et al. 2016)	−
Distance to management created waterholes	Continuous	In dry months, water becomes a critical limiting factor in the BB Complex (Thapa et al. 2023; Shah et al. 2024). Proximity to waterhole favor tiger and prey base (Kafley et al. 2016; Thapa et al., 2016)	−
Carnivore index	Continuous	There may be competition for prey, space, and cover among sympatric carnivore species. Carnivore index has no influence on tiger habitat use (Dahal et al. 2023)	−
Prey index	Continuous	Tiger abundance is determined by prey abundance (Karanth et al. 2004). High prey abundance favor tiger population (Karanth et al. 2004; Karanth et al. 2011)	+
Habitat types (Mixed Forest, Sal Forest, Tall Grassland, Riverine Forest)	Categorical	Bengal tiger is known to be a forest dwelling species. However, it is also found to be using other habitat types such as grassland and riverine forest. Thus, we selected four habitat types and modeled them to figure out tigers' preference (Sunarto et al. 2012; Rich et al. 2016)	±
Distance to human disturbance (settlements)	Continuous	Tigers prefer to inhabit undisturbed forests. Distance from settlement favor tiger and prey base (Sunarto et al. 2012; Kafley et al. 2016)	+
Detection covariates			
Camera types (Cuddeback, Panthera and Reconyx)	Categorical	Different types of camera traps may vary in their ability to detect and capture photographs of tiger as they move within the field of view. These differences can result from variations in sensor sensitivity, trigger speed, detection range, angle of coverage, and image processing capabilities. Thus, we take camera trap types as a categorical covariate for detection	±

We derived prey and carnivore indices from camera trap surveys, while remotely sensed data were used to calculate spatial covariates, including distances to waterholes, rivers, and settlements (Table 1) in the Spatial Analyst Tool extension of ArcGIS v.10.5 (ESRI, Redlands. USA). The prey index, reflecting the capture rate of prey species, was calculated as the number of photo events per 100 trap nights. The photos of a species taken within 30 min at a camera location were considered a single photo event (Carter et al. 2012). Photo events of all prey species were summed for each location to obtain the prey index (Shah et al. 2024). Similarly, the carnivore index was obtained using the same criteria for inclusion in the analysis.

We generated distance to waterholes from camera trap stations (man‐made water sources constructed by park authority as a part of habitat management). Distance to river in the study sites was calculated using ArcGIS v 10.5. The shapefile of the river was downloaded from ICIMOD (http://rds.icimod.org/Home/DataDetail?metadataId=852). Likewise, the shapefile of settlements was downloaded from Open Street map. Topographic heterogeneity was measured using the Terrain Ruggedness Index (TRI) developed by Riley et al. (1999) by using the Shuttle Radar Topographic Mission (SRTM) Digital Elevation Model (DEM) data with 90‐m resolution (downloaded from https://srtm.csi.cgiar.org/). The average value of TRI for each grid cell was used for the analysis (Thapa et al. 2019). The habitat types; mixed forest (MF), sal forest (SF), riverine forest (RF), and tall grass (TG) were classified based on visual assessments conducted by the survey team during camera trap deployment. A survey grid (2 × 2 km^2^) was assigned a specific habitat type if that habitat covered more than 50% of the grid's land area.

We standardized all continuous covariates using the *z*‐transformation method to ensure consistency and comparability (Sunarto et al. 2012; Kafley et al. 2016) and checked multicollinearity among predictors before running occupancy models (Dormann et al. 2013). Between two highly correlated variables, ecologically less meaningful covariates were excluded in the model‐building procedure (Pearson's |*r*| = > 0.7).

## 3 Results

Out of 719 camera trap locations, tigers were detected from 288 locations. Tiger photographs (Figure 1) obtained from 288 camera stations yielded naïve occupancy (habitat use) 0.40 within the Complex. Tiger detection was influenced by the types of cameras used (Table 2).

**TABLE 2:** Tiger detection probability (*p*), assuming occupancy (*ψ)* is constant, Akaike information criterion (AIC). The top‐ranked models are shown as those with ΔAIC < 2.

Models	*K*	AIC	ΔAIC	AICwt
*p*(CameraModel) psi(.)	4	5105.81	0	0.925
*p*(.) psi(.)	2	5110.85	5.04	0.075

*Note:* AIC = Akaike information criterion, AICwt. = the AIC model weight, carnivores = Carnivores index, *K* = Number of model parameters including intercepts and covariates, Habitat = types of habitats where the camera traps were deployed, Camera Model = Types of camera model used in the survey, ΔAIC is the difference in AIC values between each model and the model with the lowest AIC, *ψ* is the probability of habitat use.

We found the effect of camera trap types in tiger detection (Table 2). The Reconyx (*β =* 0.7 SE ±0.01) and Panthera (*β =* 0.224 SE ±0.1) camera traps performed better compared to Cuddeback camera traps (*β* = −2.156 SE ± 0.086) for detecting tiger. The tiger habitat use was best explained by the model that included terrain ruggedness index (tri), prey index (prey), and distance to waterholes (waterholes) in the Complex (AIC Weight = 0.77; Table 3). The top model estimated habitat use probability of tigers in BB Complex to be 0.43 (SE ± 0.0085, 95% CI: 0.414–0.448).

**TABLE 3:** Habitat use probability of tiger in Bardia‐Banke Complex. The models are shown ranked by ΔAIC values.

Models	K	AIC	delta	AICwt
*p*(CameraModel) *ψ*(prey_std + waterholes_std + tri_std)	7	4964.74	0	1
*p*(CameraModel) *ψ*(waterholes_std)	5	4992.71	27.97	0
*p*(CameraModel) *ψ*(Habitat + waterholes_std)	8	5007.62	42.89	0
*p*(CameraModel) *ψ*(tri_std)	5	5060.34	95.6	0
*p*(CameraModel) *ψ*(Habitat)	7	5062.27	97.53	0
*p*(CameraModel) *ψ*(prey_std + carnivores_std)	6	5082.09	117.35	0
*p*(CameraModel) *ψ*(prey_std)	5	5083.02	118.29	0
*p*(CameraModel) *ψ*(settlement + prey_std + river_std)	7	5085.96	121.22	0
*p*(CameraModel) *ψ*(.)	4	5105.81	141.08	0
*p*(CameraModel) *ψ*(settlement)	5	5106.55	141.81	0
*p*(CameraModel) *ψ*(carnivores_std)	5	5107.11	142.37	0
*p*(CameraModel) *ψ*(river_std)	5	5107.81	143.07	0

*Note:* AIC Akaike information criterion, AICwt. is the AIC model weight, Distanth = Distance to anthropogenic disturbances (settlement), carnivores = Carnivore index, *K* = Number of model parameters including intercepts and covariates, *p* is the probability of detection, prey = Prey index, river = Distance to river, tri = Terrain Ruggedness Index, waterholes = Distance to artificial waterholes, Habitat = types of habitat where the camera traps were deployed, ΔAIC is the difference in AIC values between each model and the model with the lowest AIC, *ψ* is the probability of habitat use.

**FIGURE 3:**
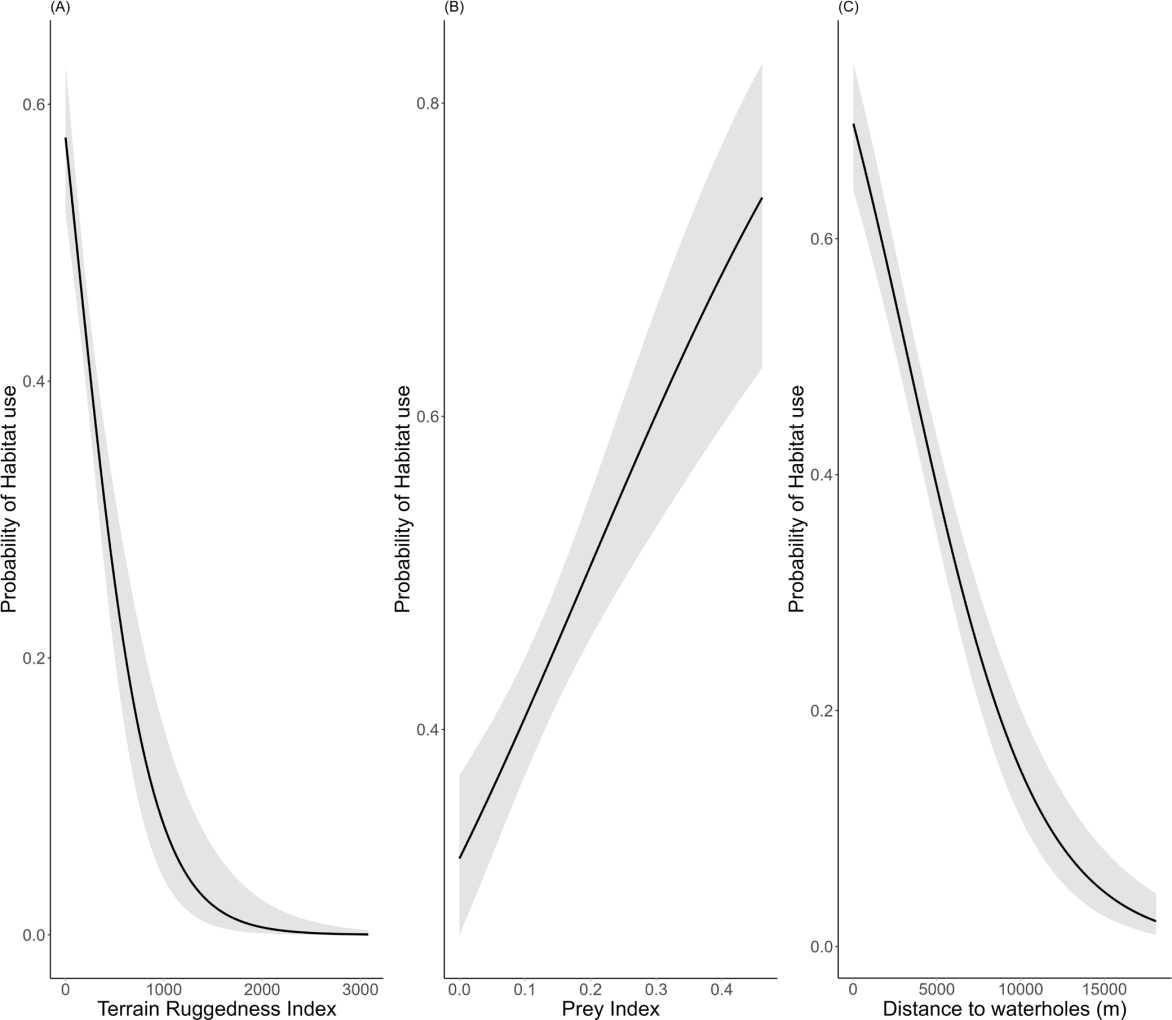
The probability of tiger habitat use in relation to the covariates: (A) Terrain Ruggedness Index, (B) Prey Index, and (C) Distance to Waterholes.

The model‐specific *β‐*coefficient values for terrain ruggedness was −0.688 (SE ± 0.1493), prey 0.103 (SE ± 0.003), and distance to waterholes −0.957 (SE ± 0.1192) (Figure 3 and Data S1). The tiger habitat use was positively influenced by prey whereas negatively associated with distance to the waterholes and terrain ruggedness in the Complex (Data S1).

**FIGURE 4:**
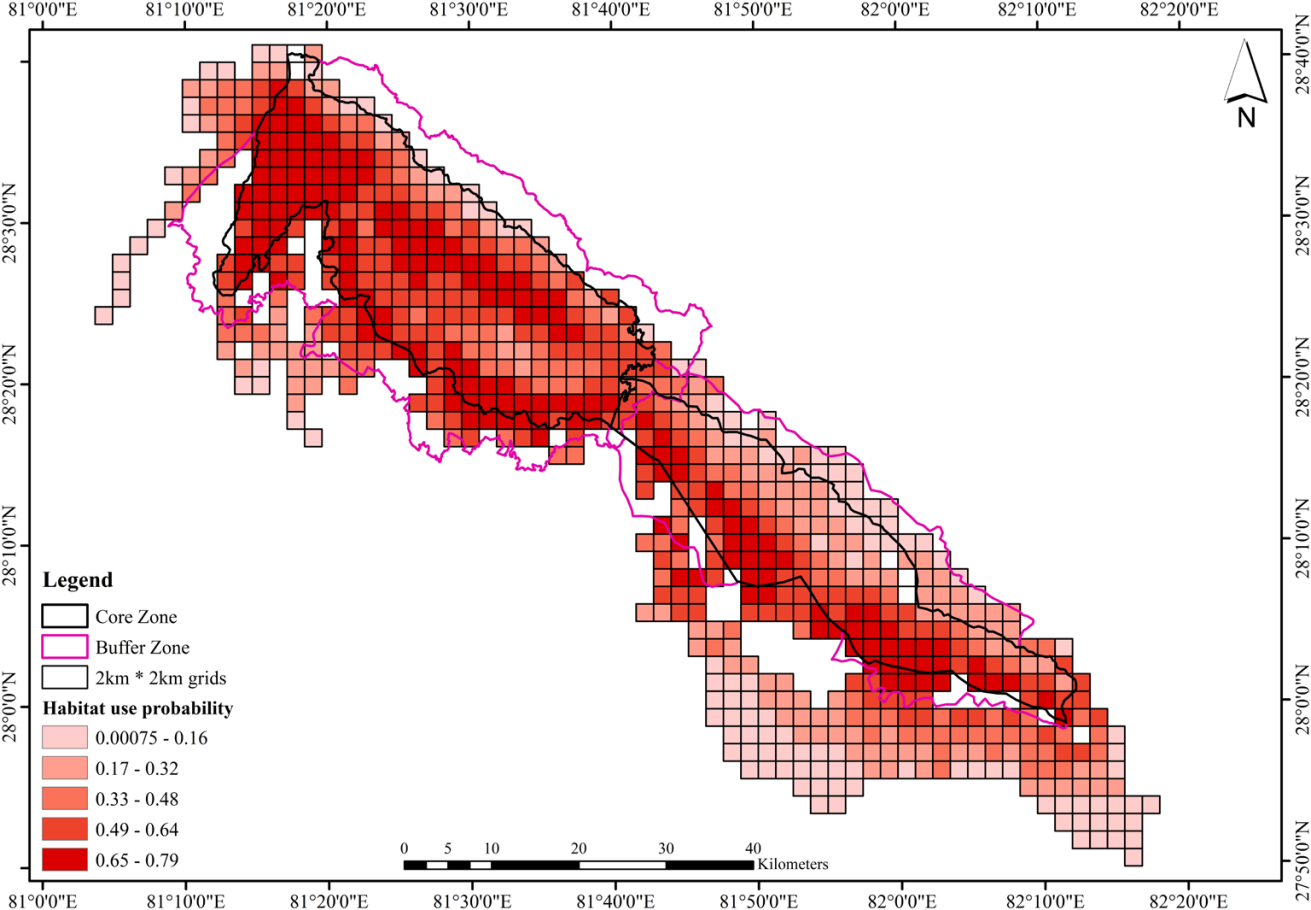
Map showing tiger habitat use in Bardia‐Banke Complex. Color gradient from light to dark indicates low to high probability of tiger habitat use.

The spatial representation of tiger habitat use (Figure 4) indicates higher habitat use in low‐elevation flat areas, near rivers and waterholes, where prey availability is relatively high.

## 4 Discussion

Our study highlights the fine‐scale habitat use of tigers in the Bardia‐Banke Complex of Nepal, a globally significant source site of tigers. Tigers were estimated to use 43% of the grids. Habitat use was primarily influenced by prey, water availability, and terrain ruggedness. The complex, one of the source sites (Walston et al. 2010) and cornerstone for tiger conservation, needs to sustain tiger recovery (Shah et al. 2024) with optimum use of available habitat.

Occupancy models are a powerful tool for species distribution modeling across medium to large spatial scales, offering robust insights into habitat use patterns. Accounting for imperfect detection in occupancy models is critical, as failure to do so can lead to inaccurate estimates (MacKenzie et al. 2002) and an underestimation of habitat use, particularly in cases of low detection—a common challenge for low‐density carnivores like tigers (Lynam et al. 2009). Tiger habitat use (*ψ* = 0.43 ± 0.016) in this Complex was notably lower compared to Chitwan National Park, Nepal (*ψ* = 0.73 ± 0.07), as reported by Kafley et al. (2016). This difference underscores potential variations in habitat quality, habitat fragmentation, frequency of forest fire, prey availability and their distribution, or human pressures including livestock grazing between the two regions. Further, the study of Kafley et al. (2016) covered core tiger habitat only, whereas our study covered the entire Complex systematically. Analysis of the tiger habitat use map (Figure 3) reveals three distinct clusters of high habitat use probability in Bardia NP: the southwest, central, and southeast. Similarly, in Banke NP, areas near the Rapti River in the south exhibit a high probability of tiger habitat use. These spatial patterns correspond with known human–tiger conflict hotspots, as reported by Kadariya et al. (2023), Dahal et al. (2023), and Paudel et al. (2024).

Our findings of tiger habitat use preferentially closer to waterholes at a fine scale align with previous studies (Kafley et al. 2016). This supports evidence highlighting water availability as a critical limiting factor for wildlife in the BB Complex, particularly in the dry season (Shah et al. 2024). Ensuring year‐round water availability at a fine spatial scale may substantially enhance tiger habitat use. Waterhole construction and wetland restoration have been a prioritized management interventions in the BB Complex to support wildlife populations (Thapa et al. 2023; BaNP 2022; BNP 2022). Tigers exhibit behaviors like ambushing prey at favored locations near water bodies (Karanth and Sunquist 2000) and stalking prey across open clearings adjacent to these resources (Thapar 1989). Proximity to water sources is particularly crucial for breeding females, as they minimize their time away from dependent cubs (Seidensticker et al. 2010). Known as water‐loving animals, tigers often frequent water sources and rivers, especially during hot sunny days (Karanth 2001).

We found decreasing tiger habitat use with increasing terrain ruggedness. The northern region of the BB Complex, characterized by hilly and rugged terrain, lacks perennial water sources and offers poor foraging habitat with limited species of prey (Figure 3). Similar patterns have been observed in Chitwan National Park (Kafley et al. 2016; Shrestha 2004). Notably, the majority of tiger detections (> 72%) were reported at elevations below 300 m (Thapa et al. 2022). Habitat use maps further corroborate these findings, showing higher tiger habitat use in the flatter southern parts of the BB Complex compared to the rugged northern part of the park.

Prey abundance has consistently been recognized as a fundamental determinant of carnivore occurrence and density (Carbone and Gittleman 2002; Fuller and Sievert 2001; Karanth et al. 2004). Across species, positive correlations between carnivore presence and prey availability have been well documented (Lamichhane et al. 2021; Thapa et al. 2021). At broader spatial scales, Karanth et al. (2004) established prey abundance as a robust predictor of tiger density. Consistent with these findings, our analysis highlights the prey index as a critical covariate influencing tiger habitat use. Prior research emphasizes that tigers demonstrate remarkable ecological resilience, provided they have access to sufficient prey, adequate habitat, and effective protection from poaching (Karanth et al. 2004; Chapron et al. 2008; Wikramanayake et al. 2011).

Our best model did not include human disturbance (settlements) as a covariate for tiger habitat use. We used distance to settlements as a proxy for human disturbance, which may not always be true. The actual disturbance events (human trespassing, resource collection, livestock grazing, etc.) at camera locations can be a realistic measure of the human disturbances.

## 5 Conclusion

Our results demonstrated that tigers used habitats unevenly across the BB Complex. Park management intervention focusing on enhancing water availability and prey base aligns well with our findings. We underscore the importance of influencing habitat covariates that determine the probability of habitat use for taking appropriate habitat‐management decisions for tiger conservation in the TAL. Periodic assessment of tiger habitat use in this globally significant source site is important to evaluate changes in spatial habitat use patterns as a measure of the effectiveness of wildlife management interventions.

## Author Contributions

**Shyam Kumar Shah:** conceptualization (lead), data curation (lead), formal analysis (lead), funding acquisition (lead), methodology (lead), writing – original draft (lead), writing – review and editing (lead). **Jhamak Bahadur Karki:** conceptualization (equal), investigation (equal), methodology (equal), supervision (lead), writing – review and editing (equal). **Balram Bhatta:** conceptualization (equal), investigation (equal), methodology (equal), supervision (equal), writing – review and editing (equal). **Naresh Subedi:** conceptualization (equal), formal analysis (equal), funding acquisition (equal), methodology (equal), supervision (equal), validation (equal), writing – review and editing (equal). **Rabin Bahadur K. C.:** data curation (equal), formal analysis (equal), investigation (equal), methodology (equal), writing – original draft (equal), writing – review and editing (equal). **Rabin Kadariya:** funding acquisition (equal), investigation (equal), methodology (equal), supervision (equal), validation (equal), writing – review and editing (equal). **Ajay Karki:** investigation (equal), supervision (equal), validation (equal), writing – review and editing (equal). **Umesh Paudel:** investigation (equal), methodology (equal), writing – review and editing (equal). **Babu Ram Lamichhane:** formal analysis (equal), investigation (equal), methodology (equal), supervision (equal), writing – review and editing (equal). **Arjun Thapa:** data curation (equal), formal analysis (equal), investigation (equal), writing – original draft (equal), writing – review and editing (equal).

## Ethics Statement

We obtained research permission from the Department of National Parks and Wildlife Conservation, Nepal (Ref no: 2443/075/76 Eco. 207; April 22, 2019). We did not carry out any experiments with live animals. Field surveys and data collection were conducted with prior approval from the department. We properly acknowledged supporting organizations for this research.

## Conflicts of Interest

The authors declare no conflicts of interest.

## Supporting information


**Data S1:** The relative strength of covariate influence (β coefficient) on tiger habitat use and detection probability in study area.

## Data Availability

Upon publication of the article, all the supporting data for obtaining the results will be made available via the dryad online data service. URL: https://datadryad.org/stash/share/EBxuUeExErm8iYyKGbFn81BacZ9tedTb‐66631IiL4.
