# Supplementary material for: Ecological Factors at Fine Spatial Scale Associated With Habitat Use by Tigers in Western Terai Arc Landscape, Nepal
Source: Ecol Evol. 2025 Mar 13;15(3):e71109. doi: 10.1002/ece3.71109 (PMC11906284; doi:10.1002/ece3.71109)
Supplement: Supplementary file 1 — Data S1: The relative strength of covariate influence (β coefficient) on tiger habitat use and detection probability in study area. [file ECE3-15-e71109-s001.docx]

**Ecological factors at fine spatial scale associated with habitat use by tigers in western Terai Arc landscape, Nepal**

Shyam Kumar Shah^1,2*^, Jhamak Bahadur Karki^1^, Balram Bhatta^1^, Naresh Subedi^3^, Rabin Bahadur K.C.^3^, Rabin Kadariya^3^, Ajay Karki^2,5^, Umesh Paudel^3^, Babu Ram Lamichhane^4,^ Arjun Thapa^6,7^*

^1^Agriculture and Forestry University (AFU), Rampur, Nepal

^2^Department of National Parks and Wildlife Conservation (DNPWC), Kathmandu, Nepal

^3^National Trust for Nature Conservation (NTNC), Lalitpur, Nepal

^4^USAID Biodiversity (Jal Jangal), Lalitpur, Nepal

^5^University of Wyoming, Laramie, Wyoming, USA

^6^Institute of Zoology, Chinese Academy of Science, Beijing, China

^7^Institute of Fundamental Research and Studies (InFeRS), Kathmandu, Nepal

*Corresponding authors: [shyamkumar_shah@yahoo.com](mailto:shyamkumar_shah@yahoo.com); [thapa.nature@gmail.com](mailto:thapa.nature@gmail.com)

**Keywords:**

Bardia - Banke Complex, Camera trapping, Habitat use, Prey index, Waterholes

**Table Supplementary information S1.** The relative strength of covariate influence (β coefficient) on tiger habitat use and detection probability in study area.

| **Category** | **Covariates** | ***β*** | **SE** | **LCI** | **UCI** |
| --- | --- | --- | --- | --- | --- |
| Occupancy | TRI | -0.688 | 0.1493 | -0.98 | -0.4 |
|  | Waterholes | -0.957 | 0.1192 | -1.19 | -0.72 |
|  | Preybase | 0.103 | 0.0031 | 0.1 | 0.11 |
| Detection | CameraModel_Cuddeback | -2.156 | 0.086 | -2.32 | -1.99 |
|  | CameraModel_Panthera | 0.224 | 0.1 | 0.03 | 0.42 |
|  | CameraModel_Reconyx | 0.7 | 0.01 | 0.68 | 0.72 |

*β is Beta coefficient; SE is Standard error); LCI is Lower confidence interval; and UCI is Upper confidence interval.*
